# SARS-CoV-2 Variants Detection Strategies in Wastewater Samples Collected in the Bangkok Metropolitan Region

**DOI:** 10.3390/v15040876

**Published:** 2023-03-29

**Authors:** Ratanaporn Tangwangvivat, Supaporn Wacharapluesadee, Papassorn Pinyopornpanish, Sininat Petcharat, Suthida Muangnoicharoen Hearn, Nattakarn Thippamom, Chadaporn Phiancharoen, Piyapha Hirunpatrawong, Phattra Duangkaewkart, Ananporn Supataragul, Chadaporn Chaiden, Wiriyachayon Wechsirisan, Nantaporn Wandee, Krongkan Srimuang, Leilani Paitoonpong, Rome Buathong, Chonticha Klungthong, Vichan Pawun, Soawapak Hinjoy, Opass Putcharoen, Sopon Iamsirithaworn

**Affiliations:** 1 Division of Communicable Diseases, Department of Disease Control, Ministry of Public Health, Muang, Nonthaburi 11000, Thailand; 2 Thai Red Cross Emerging Infectious Disease Clinical Center, King Chulalongkorn Memorial Hospital, Rama IV Road, Pathumwan, Bangkok 10330, Thailand; 3 School of Global Health, Faculty of Medicine, Chulalongkorn University, Rama IV Road, Pathumwan, Bangkok 10330, Thailand; 4 Division of Epidemiology, Department of Disease Control, Ministry of Public Health, Muang, Nonthaburi 11000, Thailand; 5 National Institute of Animal Health, Department of Livestock Development, Ministry of Agriculture and Cooperatives, Chatuchak, Bangkok 10900, Thailand; 6 Department of Medicine, Faculty of Medicine, Chulalongkorn University, Rama IV Road, Pathumwan, Bangkok 10330, Thailand; 7 Department of Disease Control, Ministry of Public Health, Muang, Nonthaburi 11000, Thailand; 8 Department of Virology, Armed Forces Research Institute of Medical Sciences, Bangkok 10400, Thailand

**Keywords:** wastewater surveillance, SARS-CoV-2 variants, COVID-19 outbreak, multiplex PCR MassARRAY

## Abstract

Wastewater surveillance is considered a promising approach for COVID-19 surveillance in communities. In this study, we collected wastewater samples between November 2020 and February 2022 from twenty-three sites in the Bangkok Metropolitan Region to detect the presence of SARS-CoV-2 and its variants for comparison to standard clinical sampling. A total of 215 wastewater samples were collected and tested for SARS-CoV-2 RNA by real-time PCR with three targeted genes (N, E, and ORF1ab); 102 samples were positive (42.5%). The SARS-CoV-2 variants were determined by a multiplex PCR MassARRAY assay to distinguish four SARS-CoV-2 variants, including Alpha, Beta, Delta, and Omicron. Multiple variants of Alpha–Delta and Delta–Omicron were detected in the wastewater samples in July 2021 and January 2022, respectively. These wastewater variant results mirrored the country data from clinical specimens deposited in GISAID. Our results demonstrated that wastewater surveillance using multiple signature mutation sites for SARS-CoV-2 variant detection is an appropriate strategy to monitor the presence of SARS-CoV-2 variants in the community at a low cost and with rapid turn-around time. However, it is essential to note that sequencing surveillance of wastewater samples should be considered complementary to whole genome sequencing of clinical samples to detect novel variants.

## 1. Introduction

As of December 2022, millions worldwide had been impacted by the coronavirus pandemic (COVID-19), in which SARS-CoV-2 is the etiologic agent [1]. In Thailand, the first confirmed case was reported in January 2020. This was followed by a sharp rise in infected patients throughout the country, in which Bangkok had the highest caseload [2]. The public health authorities of Thailand implemented various measures to control the spread of the disease, including the quarantine of international travelers immediately upon arrival to the country from April 2020 to September 2022 to restrict the importation of cases [3,4].

Based on previous reports, the genetic material of SARS-CoV-2 can be detected in the excretions of both symptomatic and asymptomatic patients [5,6]. Thus, wastewater surveillance is applicable and has been considered a crucial tool for COVID-19 surveillance, since this method helps elucidate the overall picture of COVID-19 within communities. In Italy, wastewater treatment plants were surveyed for SARS-CoV-2 RNA, and viral genomic material was detected [7]. The results were in concert with most countries, including Spain, Australia, and France, where SARS-CoV-2 genetic material could be detected in wastewater samples [8,9,10]. 

SARS-CoV-2 variants of concern (VOCs) or interest (VOIs) are variants that may evade vaccine or other pharmaceutical intervention; for example, the SARS-CoV-2 Omicron sublineage BQ.1.1 demonstrates resistance to all available monoclonal antibodies, to date [11]. Changes in the circulation of VOCs require changes in the public health response. Recently, wastewater SARS-CoV-2 variant surveillance has shown a benefit in monitoring the variants within communities, with results associated with detecting variants in clinical samples [12,13,14]. These studies highlight a significant public health use case for wastewater. However, SARS-CoV-2 RNA in wastewater may have low quantity and quality due to different degrees of degradation [15] and contamination with other pathogens [16]. Alternative methods to detect SARS-CoV-2 variants include targeted assays to increase the sensitivity, using digital RT-PCR to detect single nucleotide polymorphisms [17]. Novel targeted digital RT-PCR assays for detecting six variants have been developed and used to quantify these mutations in wastewater samples individually [12]. 

This study aims to develop and evaluate the strategy to detect SARS-CoV-2 variants from wastewater. In the present study, wastewater from fourteen study sites in Bangkok was collected from November 2020 to February 2022 and screened for the presence of SARS-CoV-2 RNA. Additionally, SARS-CoV-2 variants were further characterized in PCR-positive samples using a multiplex PCR–MassARRAY assay (PMA). We subsequently compared the variant prevalence from the wastewater to clinical specimens deposited in the GISAID, aggregated at the country level. 

## 2. Materials and Methods

### 2.1. Wastewater Sample Collection

Wastewater samples were collected from wastewater treatment tanks in Bangkok from various locations, including two hotels, one field hospital, one condominium, two fresh markets, four factories, two construction camps, and one hospital between November 2020 and February 2022. Samples were collected weekly, except where prohibited (Table 1). Approximately 200–250 mL of wastewater sample was collected simultaneously (9–11 a.m.), using a stainless steel bucket, then stored at 4 °C in sterile high-density polyethylene (HDPE) plastic and put in three-layer containers. The samples were immediately transferred to the laboratory, kept at 4 °C, and processed within 24 h. The number of samples from each area was predicated on the number of wastewater tanks available for sampling and their accessibility.

### 2.2. Wastewater Enrichment and RNA Extraction

The samples were thoroughly mixed by inversion. Then, 40 mL of mixed wastewater from 200–250 mL of the collected samples, maintained at 4 °C, was transferred to a fresh 50 mL centrifuge tube with glass beads (HiMedia, West Chester, PA, USA), then homogenized by a sample disruption instrument, FastPrep-24 5G (MP Biomedicals, Solon, OH, USA), for 60 s, at 6 m/s. The homogenized sample was centrifuged at 3000× *g* for 10 min at 4 °C to remove the precipitate. The supernatant was carefully transferred to a new tube. A ZR Urine RNA Isolation Kit (Zymo Research, Irvine, CA, USA) was used to isolate the total RNA from the samples, following the manufacturer’s protocol. The enrichment process was conducted prior to the RNA extraction. Briefly, 30–35 mL of wastewater supernatant was filtered through a 1.6 µm pore size glass fiber filtration membrane (ZRC GF Filter). Next, 700 µL of Urine RNA Buffer was applied to the filter, and the flow-through was collected in a nuclease-free tube. Following the manufacturer’s protocol, the RNA was purified with a Zymo-Spin IC Column and eluted with 50 µL of DNase/RNase-Free Water. The RNA was immediately tested for a SARS-CoV-2 real-time PCR assay and was kept at −80 °C for further analysis. 

### 2.3. SARS-CoV-2 Real-Time PCR Detection 

The presence of SARS-CoV-2 RNA was determined using the Fosun COVID-19 RT-PCR Detection Kit (Fosun, Shanghai, China) by detecting three different genes: the E gene, ORF1ab gene, and N gene. The real-time RT-PCR was performed following the manufacturer’s protocol. Briefly, 10 μL of the extracted RNA was added to a mixture of 14 μL of SARS-CoV-2 reaction reagent and 6 μL of RT-PCR enzyme. The exogenous internal positive control (IPC) RNA was included in the PCR reaction reagent to monitor the appearance of potential PCR inhibitors. Thermal cycling was performed at 50 °C for 15 min for reverse transcription, followed by 95 °C for 3 min, 5 cycles of 95 °C for 5 s to 60 °C for 40 s, and then 40 cycles of 95 °C for 5 s to 60 °C for 40 s using a Bio-Rad CFX 96 PCR instrument (Bio-Rad, Hercules, CA, USA). According to the manufacturer, samples with a PCR Ct value below 40 were considered positive.

### 2.4. Evaluation of the Wastewater RNA Extraction Protocol 

SARS-CoV-2 virus isolate (Delta variant, B.1.617.2 (hCoV-19/Thailand/CU-A21287-NT/2021/GISAID accession ID: EPI_ISL_2510689), obtained from the Department of Virology, Armed Forces Research Institute of Medical Sciences (AFRIMS), Bangkok, Thailand, was used to evaluate the wastewater enrichment extraction protocol. Eight virus concentrations (200 μL each) from 10^7^ to 1 copies/mL were spiked into 50 mL of SARS-CoV-2 negative wastewater. The RNA from the SARS-CoV-2-spiked wastewater samples was extracted as indicated above. The RNA from the virus isolate was extracted using the ZR Urine RNA Isolation Kit (Zymo Research, USA) without the above enrichment step. The extraction was conducted in triplicate for each virus concentration. The SARS-CoV-2 RNA was detected by real-time PCR, as indicated above. The PCR Ct value from the SARS-CoV-2-spiked wastewater was compared with the RNA from the direct extraction from the virus isolate in the VTM at the same viral concentration (Table 2). The limit of detection (LOD) was determined to be the last concentration where the PCR was positive. 

### 2.5. SARS-CoV-2 Variants of Concern (VOC) Detection by Multiplex PCR MassARRAY (PMA) 

The PCR-positive RNA samples with a Ct value less than 34 were further characterized for the SARS-CoV-2 variants using the multiplex PCR–nucleotide mass spectrometry technology (MassARRAY) or PMA developed in our laboratory [18]. Briefly, cDNA was generated using 8 μL of RNA extract via reverse transcription using the Superscript III First-Strand Synthesis System (Thermo Fisher Scientific, Waltham, MA, USA), following the manufacturer’s protocol. Ten primer pairs and probes were designed to detect eight specific locations on the spike gene to differentiate the Alpha (S982A, DEL69/70), Beta (DEL241/243, A701V), Delta (DEL157/158, L452R), and Omicron (E484A, S477N) variants. In addition, other targets were included as a control, including D614G for VOCs and the N gene as an optimistic gene target. 

The multiplex PCR consists of three steps. First, the amplification of 10 targets: 3 μL of cDNA was added into PCR reagents comprising 0.5 μL of PCR buffer (10×), 0.4 μL of MgCl_2_ (25 mM), 0.1 μL of dNTPs (25 mM), 0.2 μL of PCR enzyme (5 U/μL), 1 μL of amplification primer mix, and HPLC-grade H_2_O to a total volume of 6 μL. The thermocycling conditions were 95 °C for 2 min, followed by 45 cycles at 95 °C for 30 s, 56 °C for 30 s, and 72 °C for 1 min, with a final incubation at 72 °C for 5 min.

Second, to eliminate excess dNTPs from the previous step, 0.17 μL of SAP buffer (10×), 0.3 μL of SAP enzyme, and 1.53 μL of HPLC-grade H_2_O were added to the first-step PCR products to a total volume of 8 μL and incubated in thermal cycles at 37 °C for 40 min, followed by 85 °C for 5 min. 

Third, the single-base extension reaction was performed with an iPLEX Pro Reagent Kit (Agena Bioscience, San Diego, CA, USA) to hybridize and elongate the specific extension primer for each target to distinguish the mutation nucleotide from the wild type. The reaction was performed with terminator nucleotides (ddNTPs), 0.2 μL of iPLEX buffer (10×), 0.2 μL of iPLEX Terminator Mix (10×), 0.04 μL of iPLEX Pro enzyme (33 U/μL), and 0.94 μL of the extension primer mix, mixed with the second-step products, and H_2_O was added to a total volume of 10 μL. The reaction was performed at 95 °C for 30 s, followed by 95 °C for 5 s, 5 cycles of 52 °C for 5 s to 80 °C for 5 s, and then 40 cycles of 95 °C for 5 s, 52 °C for 5 s to 80 °C for 5 s, with a final extension at 72 °C for 3 min. 

Next, 29 μL of HPLC-grade H_2_O and 13 μL of clean resin were added to the extension products for desalination. Afterward, the supernatant was dispensed onto the SpectroCHIP Array through the MassARRAY Nanodispenser and scanned using a MassARRAY Analyzer. The results were analyzed using MassARRAY Typer Software (v.4.1.8.3). The mutations were distinguished with nucleotide matrix-assisted laser desorption/ionization-time of flight mass spectrophotometry (MALDI-TOF MS) based on different masses. The peaks in the mass spectrum were identified as mutations. The reported mass was generated via the MassARRAY Typer Software. The mass of nucleotides detected by the MassARRAY differentiated the wild type from the mutations. The samples with more than one variant in the wastewater specimen, wild type and mutant nucleotides were considered heterozygous.

### 2.6. Whole-Genome Sequencing by Next-Generation Sequencing (NGS)

The RNA from seven SARS-CoV-2-positive wastewater samples with a PCR Ct value lower than 26 were characterized for whole genome sequences using NGS technology. The libraries were performed using ARTIC protocol v3 and v4 primers for the viral RNA amplification [19]. The DNA library preparation was performed using an Illumina DNA Prep kit (Illumina, San Diego, CA, USA). The sequencing was achieved using a MiSeq Reagent Kit v2 (2 × 250 nucleotides) and run on an Illumina MiSeq platform (Illumina, USA). The sequencing reads were assembled by mapping to a reference sequence (SARS-CoV-2 isolate Wuhan-Hu-1) using BWA v.0.7.17 [20]. 

### 2.7. Data Analysis

We retrieved the sequence number of the SARS-CoV-2 variants of concern (Alpha, Beta, Gamma, Delta, and Omicron) in Thailand from GISAID on 7th May 2022 (Supplement Table S1). This data package contains information about the identified variants, sample locations, and collection dates [21]. This data provided information regarding each VOC’s prevalence over time and was used as a reference to compare whether the PMA results in this study corresponded with the VOC emergent period or trends in Thailand determined from the whole genome sequencing data of clinical samples.

## 3. Results

### 3.1. Country’s Reported Case Number

**Figure 1:**
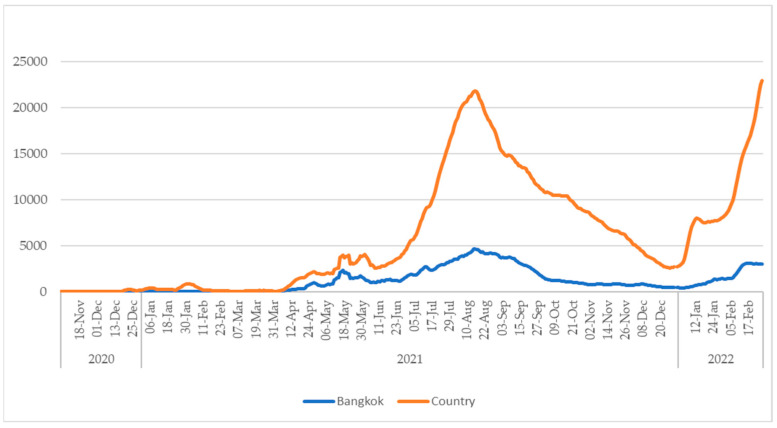
7-day moving average of the number of daily reported cases of COVID-19 in Bangkok and Thailand from November 2020 to February 2022.

From November 2020 to February 2022, Thailand reported 2,888,147 cases of COVID-19. During this time, Bangkok reported nearly 600,000 cases, accounting for approximately 20% of the national cases (Figure 1).

### 3.2. Evaluation of RNA Extraction Protocol

The efficacy of the RNA extraction from wastewater samples used in this study was assessed by comparing the sensitivity of direct viral extraction and the wastewater enrichment extraction protocols. The detection limit of the direct extraction method was 10^2^ copies/mL on all the tested genes (PCR Ct values = 32.13, 31.15, and 29.88 for the E, ORF1ab, and N, respectively), whereas the limit of detection of the wastewater extraction was 10^3^ copies/mL on all the tested genes (PCR Ct values = 32.09, 30.59, and 29.68 for the E, ORF1ab, and N, respectively). The wastewater extraction protocol showed a 10 times higher detection limit than direct extraction (Table 2). 

### 3.3. Limit of Detection of the SARS-CoV-2 RNA Assay from a Wastewater Sample

The limit of detection (LOD) of the SARS-CoV-2 RNA PCR from a wastewater sample was further calculated from the previous experiments. The LOD unit (copies per liter of wastewater) was calculated from the total copies of the spiked virus isolates at each dilution. Six concentrations of the virus isolate from 2 × 10^6^ to 20 copies were spiked into 50 mL negative wastewater specimens for final concentrations ranging from 40,000 to 0.4 copies/mL of wastewater, and the limit of detection of SARS-CoV-2 Delta in wastewater was 4 copies/mL (Table 3). 

### 3.4. SARS-CoV-2 PCR Results in Wastewater Samples from the Community

A total of 215 samples were collected from 14 sites in Bangkok from November 2020 to February 2022 (Table 1). The wastewater samples from two hotels (A and B) that were used as quarantine hotels for travelers were SARS-CoV-2 PCR-positive at the initiation of sampling and continued to be positive for 13 and 9 weeks, respectively (Table 1). The PCR Ct of N, E, and ORF1ab from hotel A was 25.48–37.76 ($\overset{-}{x}$ = 33.73), 27.38–36.44 ($\overset{-}{x}$= 33.95), and 27.32–37.35 ($\overset{-}{x}$= 32.94), respectively. The PCR Ct of N, E, and ORF1ab from hotel B was 24.5–34.7 ($\overset{-}{x}$= 29.69), 23.14–34.13 ($\overset{-}{x}$ = 30.17), and 27.24–36.6 ($\overset{-}{x}$ = 30.87), respectively.

Of the 36 wastewater samples, 19 were positive from the COVID-19 field hospital (site 3). Six samples from different wastewater tanks were collected weekly for six weeks from 22 September to 27 October 2021. During the first three weeks, four of the six, five of the six, and five of the six tested positive and decreased to one of the six, one of the six, and three of the six in weeks 4 to 6, respectively. The PCR Ct values of N, E, and ORF1ab were 24.87–33.14 ($\overset{-}{x}$ = 29.49), 23.46–34.48 ($\overset{-}{x}$ = 29.95), and 23.1–33.01 ($\overset{-}{x}$= 28.11), respectively. 

The wastewater samples from site 4, a condominium with 2000 rooms, were PCR-positive from all four samples at all four collection times. No confirmed COVID-19 patient was reported from this site at the start of the wastewater sampling. The PCR Ct values of N, E, and ORF1ab were 24.05–26.33 ($\overset{-}{x}$ = 25.06), 23.83–27.11 ($\overset{-}{x}$ = 25.20), and 22.27–25.44 ($\overset{-}{x}$ = 23.54), respectively. The viral load (estimated from the PCR Ct value) did not decrease, even after 4 weeks (Supplement Table S2).

Wastewater samples from two markets (sites 5 and 6) were collected weekly after a vendor tested positive for SARS-CoV-2. At site 5, one sample was collected from the same site for four weeks, and all four samples were positive, with PCR Ct values of N, E, and ORF1ab at 26.81–31.16 ($\overset{-}{x}$ = 28.66), 27.63–30.27 ($\overset{-}{x}$ = 28.72), and 25.28–29.65 ($\overset{-}{x}$ = 27.01), respectively. At site 6, six samples were collected from different locations in the first week and five in the second week; four of the six and two of the five samples were positive, respectively. The PCR Ct values of the wastewater samples that were positive in the first and second weeks were 24.66 and 24.98, 26.01 and 25.21, and 25.08 and 25.16 for the N, E, and ORF1ab genes, respectively. On the other hand, the PCR Ct in the first week, from the other two sites that were negative in the second week after cleaning, were 33.54 and 29.12, 34.12 and 30.24, negative, and 29.63 for the N, E, and ORF1ab genes, respectively (Supplement Table S2). 

Twenty lavatory wastewater samples were collected from the aircraft from abroad that landed at Suvarnabhumi International Airport on 29 November and 21 December 2021. Seven of the twenty samples were positive with PCR Ct values of 29.14–33.33, 30.09–34.4, and 30.0–33.8 for the N, E, and ORF1ab genes, respectively, demonstrating that at least one COVID-19 patient traveled within the aircraft.

### 3.5. SARS-CoV-2 PCR Results in Wastewater Samples from the Hospital

Twenty-four wastewater samples (*n* = 24) were collected from different areas of the hospital from December 2021 to February 2022. Fourteen water samples (58.33%) tested positive, including the COVID-19 ward (1/1), Out-Patient Department (OPD) (4/5), main wastewater treatment tank (2/2), office building (3/3), canteen building (2/2), and staff dormitory (2/4) (Table 4). The wastewater samples were negative in the In-Patient Department (IPD) building (0/5) and the surgical (Operation room, OR) building (0/2). The PCR Ct values of the SARS-CoV-2 from the OPD wastewater were 29.85–31.51, 31.35–32.62, and 29.33–31.35 for the N, E, and ORF1ab genes, respectively. As expected, the wastewater collected from the COVID-19 ward showed a lower PCR Ct value (N = 26.02, E = 26.99, and ORF1ab = 26.25) than the OPD building, indicating a higher viral load (Table 4). 

### 3.6. Screening of SARS-CoV-2 Variants in Wastewater

**Figure 2:**
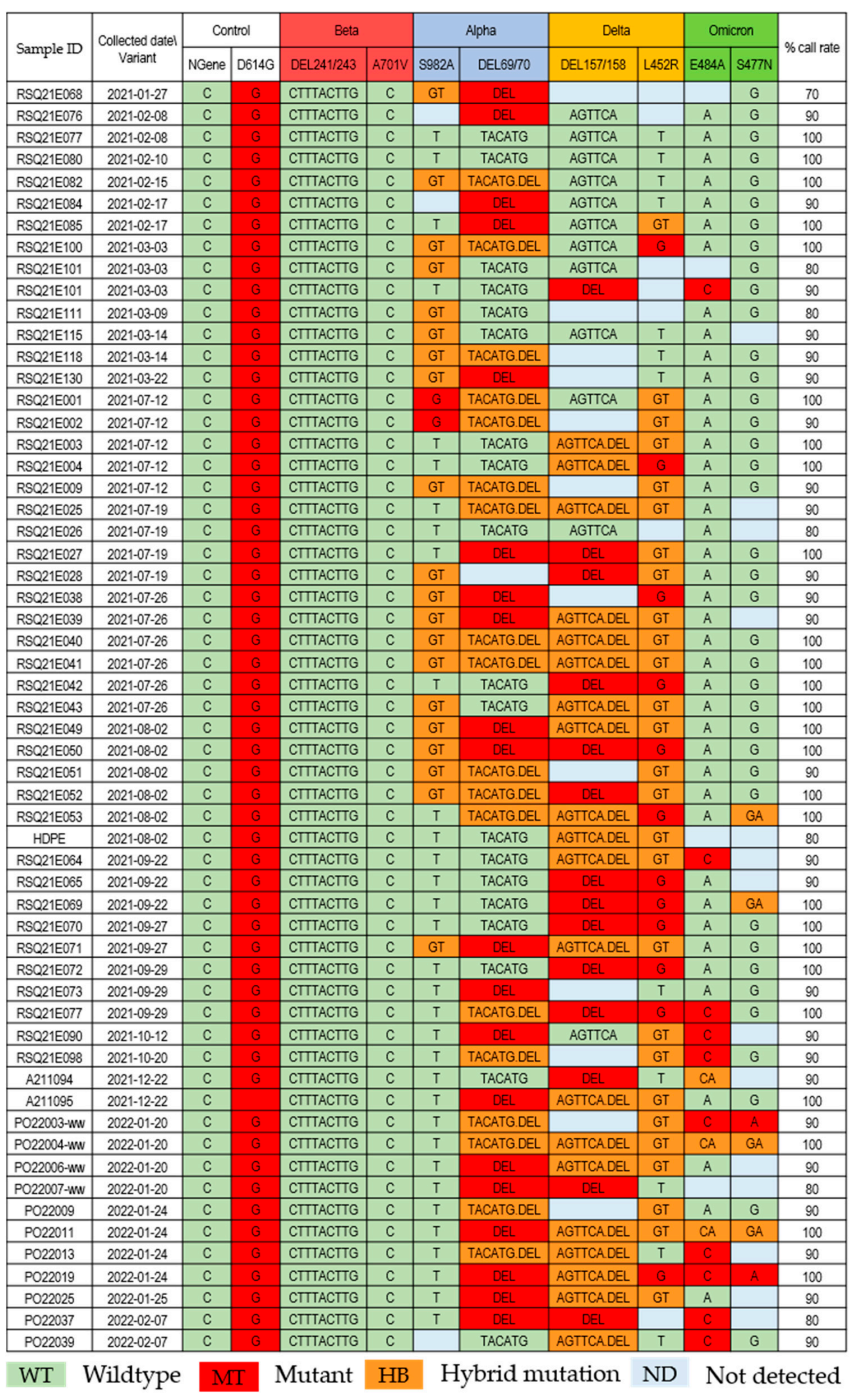
Fifty-eight wastewater sample test results were assessed for four SARS-CoV-2 variants using multiplex PCR MassARRAY assay (PMA). Variant identification by PMA at eight specific sites of the spike gene for differentiating SARS-CoV-2 variants: Alpha (S982A, DEL69/70), Beta (DEL241/243, A701V), Delta (DEL157/158, L452R), and Omicron (E484A, S477N), and two control genes (N and D614G).

Fifty-eight wastewater samples (*n* = 58) tested positive by PCR and, with a Ct value less than 34, were further tested for viral variants by a multiplex PCR MassARRAY assay (PMA). Ten PCR targets were simultaneously amplified in the same assay; eight targets were the mutation points for differentiating SARS-CoV-2 variants: Alpha (S982A, DEL69/70), Beta (DEL241/243, A701V), Delta (DEL157/158, L452R), and Omicron (E484A, S477N), and two targets were included as a control, including D614G for VOCs and the N gene as an optimistic gene target. The SARS-CoV-2 Beta variant using DEL241/243 and A701V as markers was not detected from any of the tested wastewater samples (Figure 2). The Alpha variant (S982A and DEL69/70 markers) was first detected in wastewater collected in January 2021, the same period as the first clinical sample deposited in the GISAID. The first reported case of the Delta variant in Thailand was in January 2021, and cases started to increase rapidly in May 2021, as deposited in GISAID (Supplement Table S1). Unfortunately, our study did not collect wastewater samples between March and June 2021. The Delta variant (DEL157/158 and L452R mutants) was first detected in wastewater from our study in July 2021. Mixed detection of Alpha and Delta variants was found from July to August 2021 (Figure 2). In January 2022, our study first detected the Omicron variant (DEL69/70, E484A, and S477N) in the wastewater, as well as mixed with Delta variants (Figure 2). The Omicron variant was first reported in GISAID Thailand in November 2021, and the prevalence sharply increased in January 2022 (Supplement Table S1). 

**Figure 3:**
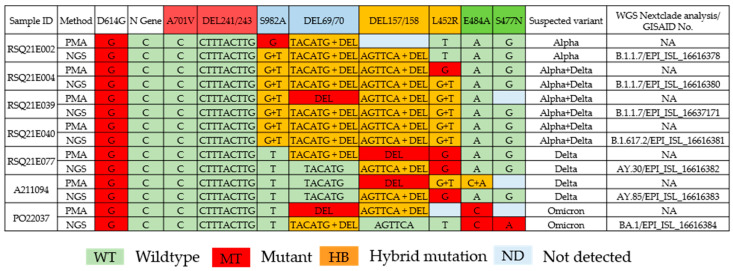
Comparison of point mutations at eight specific sites of the spike gene for differentiating SARS-CoV-2 variants detected by PMA and NGS in each wastewater specimen: Alpha (S982A, DEL69/70), Beta (DEL241/243, A701V), Delta (DEL157/158, L452R), and Omicron (E484A, S477N), and two control genes (N and D614G). The suspected variant columns indicate a possible combination of SARS-CoV-2 variants in each specimen. The SARS-CoV-2 lineage identified by Nextclade and the accession number of each NGS data point are shown in the last column.

The specificity of the PMA was validated with the whole genome sequence from next-generation sequencing of the same wastewater specimens (Figure 3). All seven whole genome sequences were deposited into GISAID with the following accession numbers: EPI_ISL_16616378, EPI_ISL_16616380, EPI_ISL_16637171, EPI_ISL_16616381, EPI_ISL_16616382, EPI_ISL_16616383, and EPI_ISL_16616384. In addition, the target point mutations were identified from the Sequence Alignment Map file using Unipro UGENE [22] to extract the nucleotide coverage per base. The suspected SARS-CoV-2 variants of all seven samples concluded from the eight specific point mutations reported by the PMA and the NGS are the same (Figure 3). 

## 4. Discussion

Wastewater surveillance is a cost-effective tool to survey outbreaks. Wastewater surveillance is used in many countries to track COVID-19 circulation and SARS-CoV-2 variants at the community level [12,13,14,23]. In combination with other indicators, wastewater surveillance is beneficial in targeting the appropriate COVID-19 responses and interventions by providing an early indication (4–7 days) of changes in the incidence and levels of virus circulation [24,25,26]. 

Our study demonstrates a simple and easy method for detecting SARS-CoV-2 RNA and its variants from wastewater specimens. There have been many surveillance studies to detect viruses from wastewater since 2020 that have used different methods of sample-collection, enrichment, and purification of viral RNA [27]. The studies that require special equipment, for example, using the ultracentrifugation at the enrichment step to increase the viral RNA copy, raise limitations in conducting wastewater surveillance in resource-limited countries. Before extraction, this study used commercially available extraction kits (Zemo Research, Irvine, CA, USA) to enrich the viral RNA on a filter. Our study’s LOD of wastewater SARS-CoV-2 detection protocol is four copies/mL (Table 3). Similar results on the LOD of wastewater PCR detection have been reported by Thongpradit et al.; the LOD for three gene targets, N, ORF1ab, and S, was 1.63, 1.20, and 1.51 copies/mL, respectively. The study demonstrates the ability to detect SARS-CoV-2 RNA in wastewater, even with only one patient in the COVID-19 isolation ward [28]. 

To demonstrate the practicality of our method, eight different wastewater sampling locations (fourteen sites) were included in this study: a hotel, market, factory, construction camp, hospital, field hospital, condominium, and aircraft. Positive samples were found at all the sites, including the sewage lavatory from the aircraft, where access to clinical samples is extremely limited. The sampling frequency varied between 1 and 16 times at each sampling site. The viral copy was not determined in this study, but we used the PCR Ct value to estimate the amount of the virus. The PCR Ct value varied from 23.31–37.76, 22.19–36.44, and 20.64–37.35 when detected in N, E, and ORF1ab, respectively. Of the 102 samples, 86 were PCR-positive for all three genes, 15 were positive for two genes, and one was positive for only the ORF1ab gene (PCR Ct value = 33.8). This result is comparable to detecting SARS-CoV-2 RNA in clinical samples, in which the assay’s sensitivity is increased when multiple gene targets are used for detection [29]. The absence of SARS-CoV-2 RNA in the wastewater samples from the market in the second week demonstrates that interventions such as closing and cleaning help to control and eliminate the virus from the system. The continuing SARS-CoV-2 RNA-positive results from the wastewater collected from the condominium confirm that there were COVID-19 patients in the building during the sampling period, even though there were no reports of COVID-19 cases.

Aircraft wastewater surveillance can form part of a broader strategy for public health surveillance at national borders [30]. Aircraft wastewater samples were collected from 20 flights in our study, and seven samples were positive. The PCR Ct values of the N, E, ORF1ab range were 29.14–33.33, 30.08–34.4, and 30.0–33.8, respectively. These PCR Ct value levels were similar to the wastewater tested from the hospital when four patients were admitted (N = 31.94, ORF1ab = 33.89, and S = 32.01), as reported previously [28]. This finding revealed that COVID-19-infected travelers entered the country despite strict travel regulations from November to December 2021. SARS-CoV-2 RNA was not detected in the wastewater collected from clean areas, such as the non-COVID-19 IPD and OR wards. The wastewater study at the hospital site demonstrated that the COVID-19 prevention measures used for the clean areas, such as the non-COVID-19 IPD and OR wards, are proper for screening and excluding COVID-19-infected patients. The hospital infection control measures included collecting the contact history for the seven days before admission, the temperature measurement, and a SARS-CoV-2 RT-PCR test from each patient before hospital admission. The patient caregiver was also tested for SARS-CoV-2 by RT-PCR on the first day and every subsequent seven days. SARS-CoV-2 detection in wastewater can help monitor the effectiveness of the control measures. 

The study also highlights that genomic surveillance of SARS-CoV-2 in wastewater could track clinically relevant mutations, which might be underrepresented in clinical sequencing data. The wastewater samples contained low viral loads of SARS-CoV-2 because they were diluted by the massive volume of water and low viral RNA quality, due to degradation from the treatment process [15,16]. Testing of the SARS-CoV-2 viral variants by the whole genome was therefore limited; the limit of detection for successful whole genome sequencing is a PCR Ct value less than 26 [18]. The PCR Ct values from the wastewater samples in this study and others [28,31] were mostly higher than 30. An alternative approach for SARS-CoV-2 variant detection is applying target amplification and detecting short genome sequences characteristic of genome variants [17,32,33,34] using digital PCR technology. Our study used multiplex PCR to amplify and then used MassARRAY technology (PMA) to detect the short spike gene fragments in wastewater. This technology allows the detection of wild type and mutant nucleotides for the SARS-CoV-2 variant in the same reaction. It has been successfully used to simultaneously identify SARS-CoV-2 variants from biological specimens on nine mutation sites on a sample with a PCR Ct lower than 27 [35]. Recently, PMA was developed to detect 24 mutation targets simultaneously in one reaction [18]. The sensitivity of each target varied from 10 to 1000 copies/mL. 

**Figure 4:**
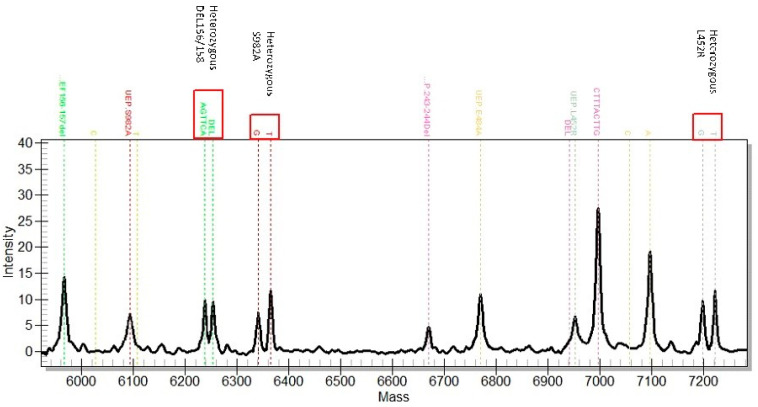
The mass spectrum of SARS-CoV-2 spike genes from wastewater specimen. Two SARS-CoV-2 variants were identified. Heterozygous peaks of three targets are shown: DEL156/158, S982A, and L452R. DEL156/158 and L452R are the specific markers for Delta, and S982A and DEL69/70 are the specific markers for the Alpha variant. UEP—unextended primer.

In this study, primers and probes were designed to detect four significant VOCs from January 2021 to February 2022, including Alpha, Beta, Delta, and Omicron variants, using PMA. These included eight unique mutation points and two control sites (the N gene as the SARS-CoV-2 positive control and D614G as the VOC site). The overall sensitivity (call rate) of 58 wastewater samples was 100% (*n* = 26), 90% (*n* = 25), 80% (*n* = 6), and 70% (*n* = 1) (Figure 2). A mixture of genomic material (wild type and mutant) of multiple SARS-CoV-2 variants present in wastewater samples can be distinguished by the spectrum of a mass detected from MassARRAY (Figure 4). MassARRAY technology allows the detection of heterozygous mutation points. It is an assay to detect coinfection and multiple infections from the same sample in one reaction. The process takes 8 h, and the reagent cost is cheaper than the whole genome sequencing assay [18]. PMA is the method of choice for variant identification in wastewater samples, in which the pooled sample can contain multiple virus strains (variants). In contrast, the presence of multiple SARS-CoV-2 variants could not be determined from the consensus whole genome sequence obtained from the NGS technology in this study, as only the major mutation was called for each position. Therefore, all the point mutations found in each position were extracted directly from the variant calling file (Figure 3).

COVID-19 cases infected with the Beta variant were rare in Thailand (112 reported in the GISAID), so it is not unexpected for the wastewater testing to reveal no infection. The association between the detection of the SARS-CoV-2 Alpha variant in wastewater and the occurrence of the variant in clinical specimens in January 2021 from our study was consistent with the results from the USA, Canada, Israel, and Europe [12,13,33,34,35]. The SARS-CoV-2 variant results from wastewater collected from January 2021 to February 2022 indicated the replacement of consecutive variants in circulation over time (Figure 2). The Delta variant was first detected in wastewater in July 2021, while the sharp rise of the Delta variant in the clinical samples deposited in GISAID was found in May 2021; unfortunately, there were no samples collected and tested during April and June 2021 in our study. The SARS-CoV-2 Omicron variant was first reported in a patient in Thailand in November 2021, but it was first found in wastewater in January 2022. Two wastewater samples from the community were collected in November 2021. The disagreement between the variant’s presence in the clinical and wastewater samples from our study was due to the low number of samples (the variant data were received from 58 of 104 positive samples) and inconsistency of the sampling time and period.

The limitations of this study are the inconsistency of the sampling number and the frequency of the sampling time. In addition, the comparable data of the SARS-CoV-2 variant detection in wastewater samples and GISAID data from many laboratories in Thailand reveal an alternative method for monitoring the relative prevalence of variants in each community, especially in a location where randomized sequencing from clinical specimens might be a challenge. Indeed, there are limitations to detecting new pathogens using the PMA method. However, it is essential to note that sequencing surveillance of wastewater samples should be considered complementary to whole genome sequencing of clinical samples. Therefore, it is necessary to monitor the whole genome sequence from patients or wastewater, and then update any new mutation points in the PCR and PMA panels. The results will be further implemented as the basis for COVID-19 outbreak and variant screening in communities that will work adequately in Thailand. 

## 5. Conclusions

Our findings demonstrate a simple method for SARS-CoV-2 detection from wastewater collected from various settings. It can be used to monitor the circulation of the SARS-CoV-2 virus in the community and assess the infectious control measures in a COVID-19-free environment, such as an operating room, non-COVID-19 ward, or aircraft. In addition, it can be expanded to other contagious infectious diseases such as gastrointestinal tract infection or Mpox. The samples for wastewater surveillance should be collected continuously (at least two specimens per site) and consistently. In addition, the primers for multiplex PCR should be updated frequently to avoid missed SARS-CoV-2 variant identification. 

## Figures and Tables

**Table 1:** Overview of sampling sites, sampling dates, number of sampling times and frequency, number of samples collected each time, number of the SARS-CoV-2 RT-PCR-tested, and positive samples.

Site No.	Site Character	Samples Collection Period		Sampling Time (Frequency) [No. of Sample/Time] *	No. Tested Sample	No. Positive Sample
		Start Date	End Date			
1	Hotel A	23 November 2020	22 March 2021	16 (weekly) [2]	32	12 ^(1)^
2	Hotel B	19 January 2021	24 March 2021	10 (weekly) [3]	30	11 ^(2)^
3	Field hospital	22 September 2021	27 October 2021	6 (weekly) [6]	36	19
4	Condominium	12 July 2021	2 August 2021	4 (weekly) [4]	16	16
5	Market A	12 July 2021	2 August 2021	4 (weekly) [1]	4	4
6	Market B	14 March 2021	22 March 2021	2 (weekly) [5,6]	11	6
7	Factory A	11 October 2021	9 November 2021	1 [1]	7	1
8	Factory B	18 October 2021	18 October 2021	1 [1]	1	0
9	Factory C	12 July 2021	2 August 2021	4 (weekly) [4]	16	1
10	Factory D	12 July 2021	2 August 2021	4 (weekly) [3]	12	9
11	Construction Camp A	25 November 2021	25 November 2021	1 [2]	2	0
12	Construction Camp B	27 September 2021	27 September 2021	1 [2]	2	2
13	Hospital	22 December 2021	23 February 2022	1–2, 14 sites [1]	25	14
14	Aircraft	29 November 2021	21 December 2021	20 planes [1]	20	7
	TOTAL				215	102

* Collection volume was 200–250 mL. ^(1)^ The wastewater samples tested negative on weeks 2, 3, 5, 6, 8, 9, 14–16. ^(2)^ The wastewater samples tested negative on weeks 6 and 10.

**Table 2:** SARS-CoV-2 RT-PCR results of Delta virus isolate and wastewater samples spiked with Delta virus separated at eight dilutions from 1 to 10^7^ copies/mL.

Virus Concentration	Direct Extraction PCR Ct Value *			Spike into Wastewater Sample PCR Ct Value *		
Copies/mL	E	ORF1ab	N	E	ORF1ab	N
10^7^	16.23	15.52	14.24	18.90	17.88	16.81
10^6^	20.08	19.38	18.15	23.16	22.27	21.10
10^5^	23.25	22.58	21.29	26.42	25.72	24.46
10^4^	27.07	26.65	25.11	28.35	27.94	26.43
10^3^	30.86	30.45	28.75	32.09	30.59	29.68
10^2^	32.13	31.15	29.88	-	-	-
10	-	-	-	-	-	-
1	-	-	-	-	-	-

* Mean of triplicate assays.

**Table 3:** The PCR Ct results of six wastewater samples spiked with Delta virus isolate with a final viral load between 40,000 to 0.4 copies/mL.

Total Viral Copies	LOD Copies/mL	PCR Ct Value *			
		E	ORF1ab	N	Internal Control
2 × 10^6^	4 × 10^4^	18.90	17.88	16.81	18.53
2 × 10^5^	4 × 10^3^	23.16	22.27	21.10	18.73
2 × 10^4^	4 × 10^2^	26.42	25.72	24.46	18.64
2 × 10^3^	40	28.35	27.94	26.43	18.56
2 × 10^2^	4	32.09	30.59	29.68	18.89
20	0.4	ND	ND	ND	18.84

* Mean of triplicate assays. ND means the sample was not detected by PCR assay.

**Table 4:** SARS-CoV-2 RT-PCR results in wastewater samples collected from various sites at the hospital in Bangkok from December 2021 to February 2022.

Sample No.	Collected Date	Collection Site’s Name/Building Function	PCR Results	PCR Ct Value		
				E	N	ORF1ab
A211098	24 December 2021	C1.1A/non covid IPD + OPD	Not detected	-	-	-
A211099	24 December 2021	C1.1B/non covid IPD + OPD	Not detected	-	-	-
PO22001	20 January 2022	C1.1A/non covid IPD + OPD	Not detected	-	-	-
PO22002	20 January 2022	C1.1B/non covid IPD + OPD	Not detected	-	-	-
PO22023	25 January 2022	C1.2/non covid IPD + OPD	Not detected	-	-	-
PO22005	20 January 2022	C1.3/OR	Not detected	-	-	-
PO22035	7 February 2022	C1.3/OR	Not detected	-	-	-
PO22006	20 January 2022	C1.4/OPD	Detected	31.94	30.41	29.33
PO22039	7 February 2022	C1.4/ OPD	Detected	31.358	29.857	30.78
PO22019	24 January 2022	C1.5/OPD	Detected	32.625	31.508	31.346
PO22043	23 February 2022	C1.5/OPD	Detected	30.6	28.68	30.18
PO22021	25 January 2022	C1.6/OPD	Not detected	-	-	-
PO22037	7 February 2022	C1.7/COVID ward	Detected	26.994	26.023	26.247
A211094	22 December 2021	C1.8A/main treatment tank	Detected	27.076	24.261	26.962
A211095	22 December 2021	C1.8B/ main treatment tank	Detected	32.882	31.167	33.869
PO22003	20 January 2022	C2.1A/office building	Detected	31.25	29.3	28.76
PO22004	20 January 2022	C2.1B/office building	Detected	34.11	31.83	33.09
PO22009	24 January 2022	C2.1A/office building	Detected	34.139	31.057	33.58
PO22007	20 January 2022	C2.2/canteen	Detected	31.44	32.68	32.89
PO22025	25 January 2022	C2.2/canteen	Detected	31.035	30.125	30.736
PO22011	24 January 2022	C3.1/staff dormitory	Detected	30.489	27.536	29.185
PO22013	24 January 2022	C3.2/staff dormitory	Detected	34.042	30.549	30.994
PO22015	24 January 2022	C3.3/staff dormitory	Not detected	-	-	-
PO22017	24 January 2022	C3.4/staff dormitory	Not detected	-	-	-

IPD = In-Patient-Department; OPD = Out-Patient-Department; OR = Operation room.

## Data Availability

Not applicable.

## Supplementary Materials

The following supporting information can be downloaded at: https://www.mdpi.com/article/10.3390/v15040876/s1, Table S1: Number of SARS-CoV-2 variants of concern from Thailand deposited in GISAID, data accessed on 7 May 2022; Table S2: PCR results of SARS-CoV-2-positive wastewater specimens.
